# Supplementary material for: DNA Damage Response during Replication Correlates with CIN70 Score and Determines Survival in HNSCC Patients
Source: Cancers (Basel). 2021 Mar 10;13(6):1194. doi: 10.3390/cancers13061194 (PMC7998578; doi:10.3390/cancers13061194)
Supplement: Supplementary file 1 [file cancers-13-01194-s001.pdf]

Supplementary Materials

DNA Damage Response During Replication Correlates with CIN70 Score and Determines Survival in HNSCC Patients

Ioan T. Bold[1,+,] Ann-Kathrin Specht[1,+], Conrad F. Droste[2], Alexandra Zielinski[1], Felix Meyer[1], Till S. Clauditz[4], Adrian Münscher[5], Stefan Werner[6], Kai Rothkamm[1], Cordula Petersen[7], and Kerstin Borgmann[1,*]

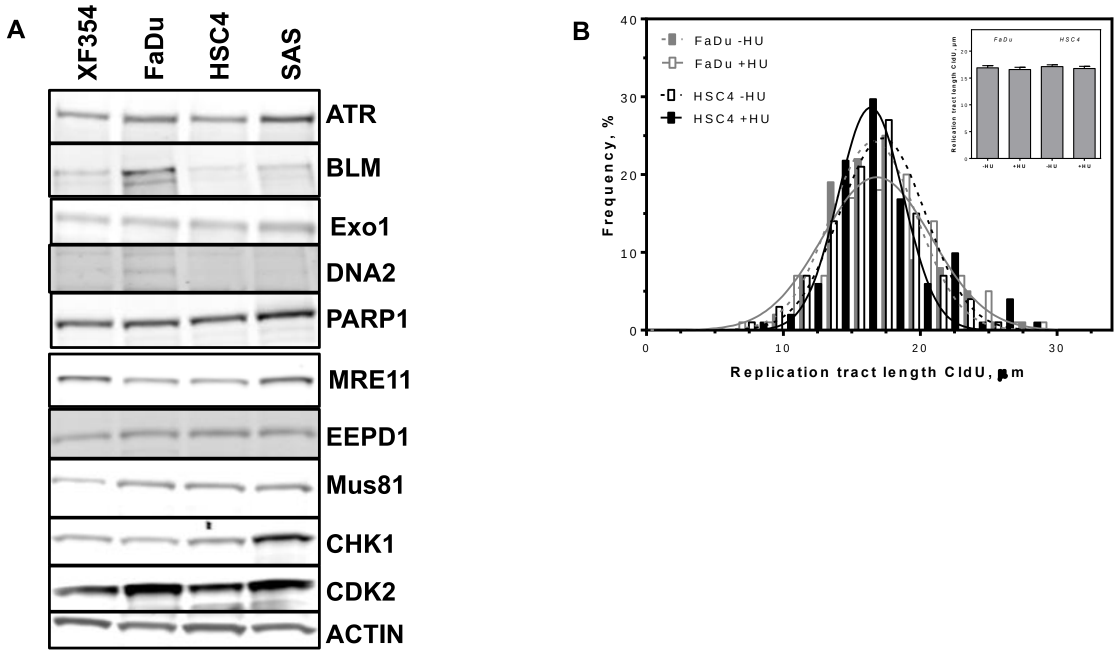

Figure S1. (**a**) Protein expression of ATR, BLM, Exo1, DNA2, PARP1, MRE11A, EEPD1, Mus81, CHK1, CDK2 and Actin in the two radiosensitive and radiorestant cell lines; (**b**) Frequency of replication tract lengths after treatment with hydroxy urea.

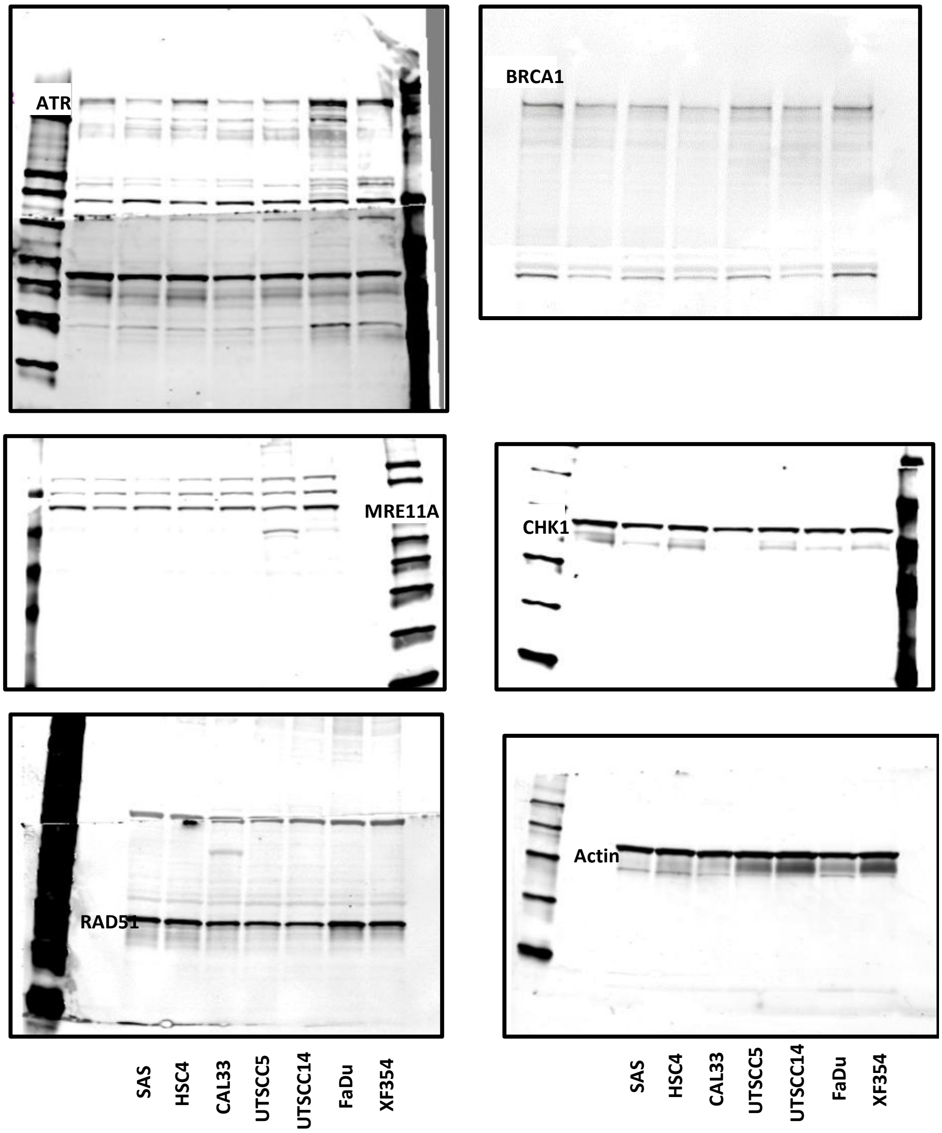

Figure S2. Western Blot Figure 2C

Figure 4F

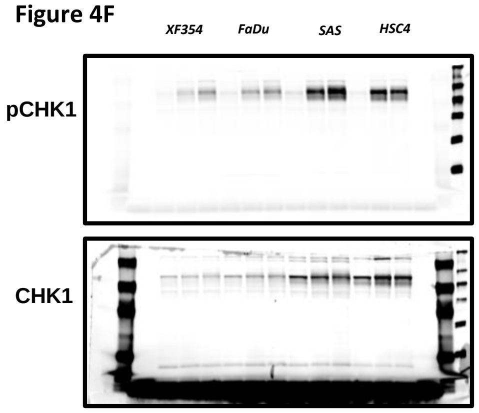

Figure 5A

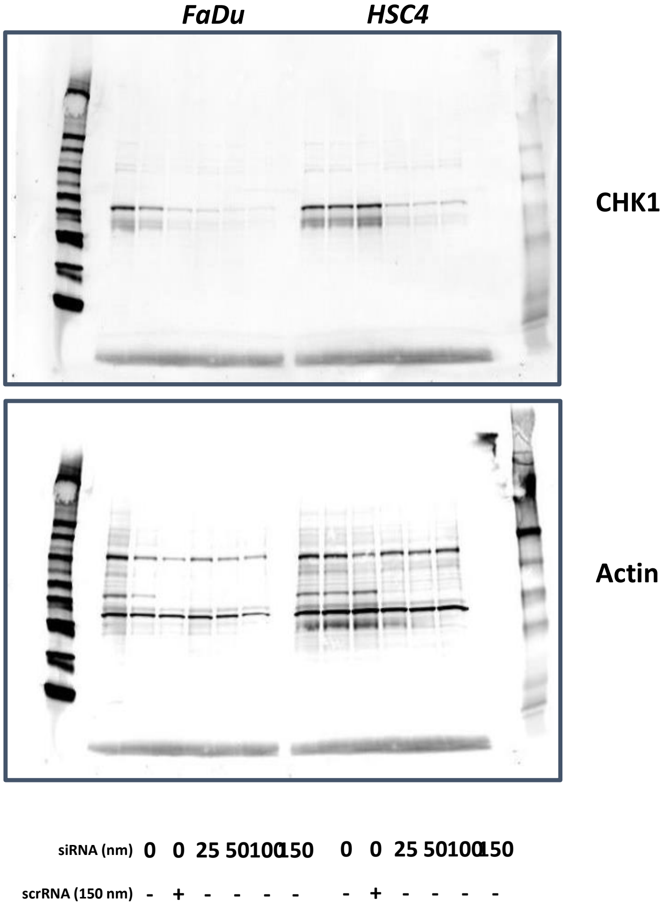

Figure S3. Western blot Figure 4F and Figure 5A

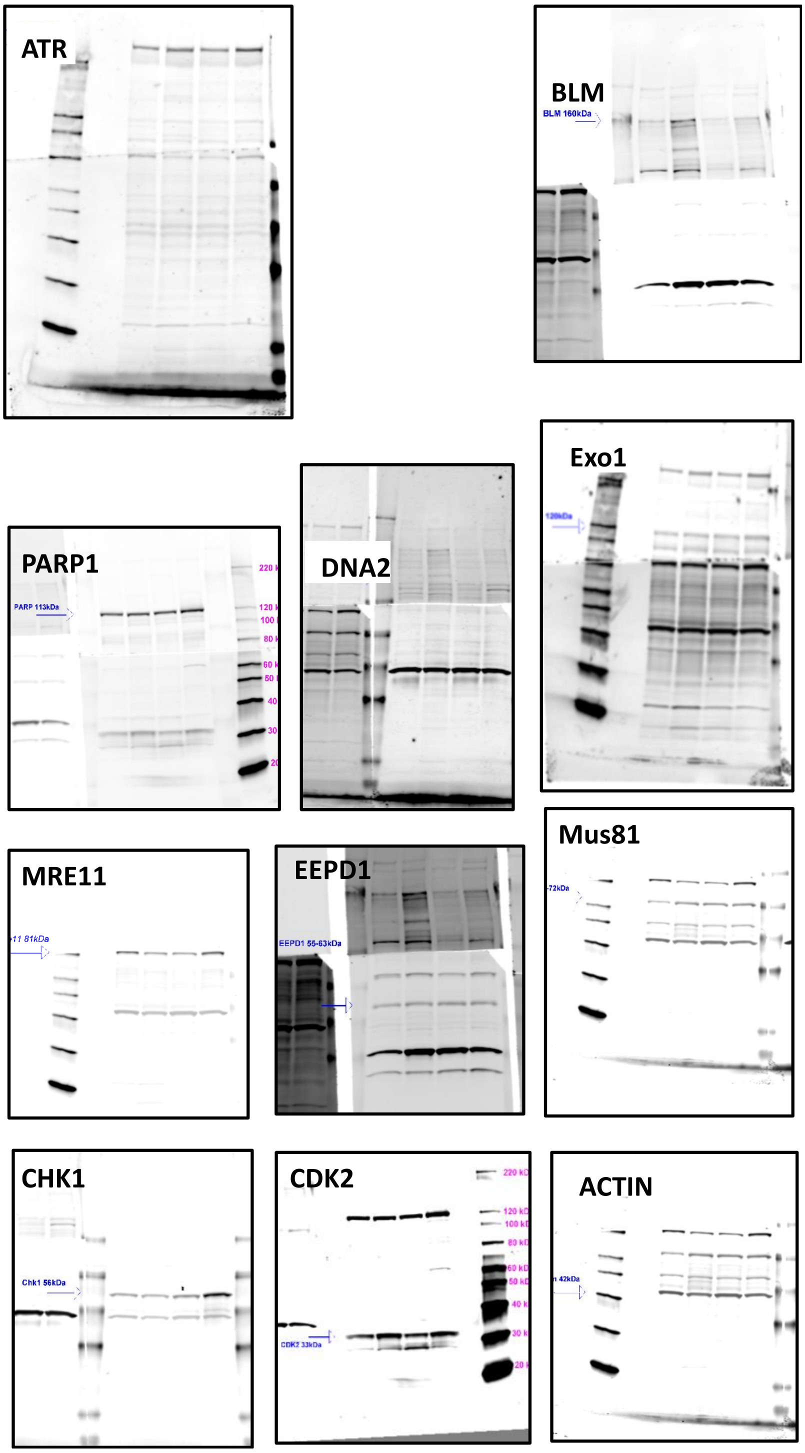

Figure S4. Western blot Figure S1A

© 2020 by the authors. Licensee MDPI, Basel, Switzerland. This article is an open access article distributed under the terms and conditions of the Creative Commons Attribution (CC BY) license (http://creativecommons.org/licenses/by/4.0/).
